# Evaluation of Micronucleus Count in Oral Squamous Cell Carcinoma (OSCC) Newly Diagnosed Patients Compared to Previously Treated Ones: A Cytologic Study -

**DOI:** 10.31661/gmj.vi.3968

**Published:** 2025-12-15

**Authors:** Marjan Mohammadi, Noushin Jalayer Naderi, Ata Garajei, Seyed Masoud Sajedi

**Affiliations:** ^1^ Department of Oral and Maxillofacial Medicine, Faculty of Dentistry, Shahed University, Tehran, Iran; ^2^ Department of Oral and Maxillofacial Pathology, Faculty of Dentistry, Shahed University, Tehran, Iran; ^3^ Department of Oral and Maxillofacial Surgery, School of Dentistry, Tehran University of Medical Sciences, Tehran, Iran; ^4^ Department of Head and Neck Oncologic and Reconstructive Surgery, The Cancer Institute, School of Medicine, Tehran University of Medical Sciences, Tehran, Iran

**Keywords:** Mouth Mucosa, Micronucleus Assays, Oral Squamous Cell Carcinoma, Cytology, Screening

## Abstract

**Background:**

Early detection and monitoring of genomic damages are vital for improving therapeutic outcomes. Quantification of micronuclei in exfoliated buccal mucosa cells has emerged as a reliable biomarker for assessing genomic alterations and cytogenetic damage in precancerous and cancerous conditions.

**Materials and Methods:**

This study evaluated exfoliated buccal cells from two groups of OSCC patients: seventeen newly diagnosed individuals who hadn’t yet undergone OSCC treatments and seventeen patients assessed at least six months after treatments. Micronuclei were identified and quantified in the cytology samples, and statistical analyses including the T-test, Mann–Whitney U, Kruskal–Wallis, and Spearman’s correlation tests were applied at a significance threshold of P0.05 to compare modalities between groups.

**Results:**

The newly diagnosed group exhibited a mean micronucleus frequency of 0.028±0.013 per 10³ cell, whereas the treated group demonstrated a significantly lower mean frequency of 0.016±0.020 per 10³ cell (P=0.03). Further stratification of treated patients by intervention type (surgery alone, surgery combined with radiotherapy, and surgery followed by radiotherapy and chemotherapy) yielded mean counts of 0.006±0.003 per 10³ cell, 0.014±0.010 per 10³ cell, and 0.026±0.025 per 10³ cell, respectively. These variations did not reach statistical significance (P=0.29).

**Conclusion:**

The findings show that treatment reduces cytogenetic damage, as reflected by diminished micronucleus formation. Consequently, micronucleus assessment in buccal mucosa cells may serve as a noninvasive, cost-effective tool for monitoring therapeutic efficacy and predicting the recovery process in OSCC patients.

## Introduction

Oral cavity squamous cell carcinoma cells, due to exposure to various genotoxic agents, often exhibit errors in chromosomal segregation, leading to the formation of lagging chromosomes or chromosomal fragments that detach from the forming nuclei during anaphase. These fragments appear in the cytoplasm of daughter cells as multiple secondary nuclei, which are smaller than the main nucleus and are called micronuclei [1][2]. Micronuclei are extranuclear cytoplasmic bodies with a diameter less than one third of the main nucleus, formed as a result of chromosomal damage. These bodies have staining intensity, texture, and structure similar to the nucleus but lack a direct connection to it, making them identifiable in cytological samples [3][4].

The average frequency of micronuclei in cells of the general population has been reported 0 to 0.9%. The increased count of micronuclei can be interpreted as a chromosomal change [5] and may indicate a higher likelihood of malignancy especially in tobacco smokers [1][6][7]. According to previous studies, the examination of micronuclei count in exfoliated cell samples from the buccal mucosa can be used as a biomarker for investigating genomic alterations and cytogenetic damage in precancerous and cancerous conditions of oral cavity [8][9].

The average micronucleus count has been reported higher in patients with OSCC than in those with leukoplakia and healthy controls [9]. It also has been shown that the number of micronuclei in patients with OSCC is significantly higher than healthy cases [2]. These findings suggest that micronucleus counts offer a valuable approach both for prognostic assessment of neoplastic lesions and for distinguishing between precancerous and cancerous samples. OSCC is a life-threatening disease that can lead to death. Treated patients should undergo long-term follow-ups to investigate the possibility of recurrence and re-biopsy if necessary. Repeated biopsies can be painful and unbearable for the patient undergoing treatment. So far, no method with appropriate sensitivity has been introduced to monitor and follow OSCC patients; thus, finding an available, non-invasive method is of vital importance. Since micronuclei can be detected under cytogenetic changes, it seems that the examination of micronucleus count can be proposed as an appropriate tool for such purpose. The aim of this study was to evaluate the micronucleus count in patients with newly diagnosed OSCC compared to treated patients to clarify its utility in monitoring therapeutic outcomes.

## Materials and Methods

This study was conducted at the Cancer Institute of Imam Khomeini Hospital, affiliated with Tehran University of Medical Sciences, Tehran, Iran, between 2023 and 2025. A total of thirty-four participants were enrolled, comprising seventeen patients newly diagnosed with oral squamous cell carcinoma (OSCC) and seventeen individuals with a history of the disease who had undergone treatment. The protocol of the study was approved by the Ethical Committee on Biological Researches of Shahed University and registered as IR.SHAHED.REC.1402.088.

Inclusion criteria required participants to have no history of infectious or immunological diseases and no occupational exposure to pesticides or related industries. For the treated group, a minimum interval of six months since the last treatment was mandatory. Exclusion criteria encompassed current tobacco or alcohol use and, except for the treated group, any radiotherapy received within the preceding year.

After obtaining informed consent, demographic data (including age, gender, lesion site, and disease duration) were recorded. In the treated group, the therapeutic modalities including surgery, radiotherapy, and chemotherapy, as well as the time elapsed since treatment completion were also documented. Each participant was assigned a unique code under which all information was catalogued.

**Figure-1:**
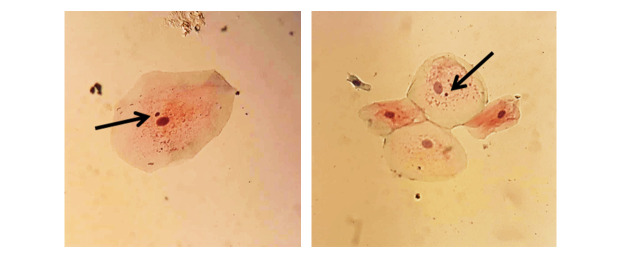
Figure 1. Micronucleus in exfoliated buccal cells of an OSCC patient (PAP stain, x400)

Cytological sampling of the buccal mucosa was performed as follows: Subjects first rinsed their mouths twice with running water. Mucosal cells were then gently collected with a disposable plastic brush and immediately smeared onto clean glass slides. The slides were fixed using a cytology fixative spray (NamiraCyte, Bahar Afshan, Iran) and stained using the Papanicolaou (PAP stain) method. Micronuclei were counted in a total of one thousand cells per sample under a light microscope (Zeiss, Germany) at 400× magnification. Only isolated cells with clear margins and well-defined nuclei were evaluated. Micronuclei were identified as follows: they exhibited the same coloration as the nucleus, were adjacent to the main nucleus, measured between one-third and one-fifth of the nucleus diameter, and were devoid of any connection to the nucleus [4]. (Figure-1)

The average number of micronuclei per sample was calculated for each group. Statistical comparisons between the newly diagnosed and treated groups were performed using the T-test and the Mann-Whitney U test, while differences among treatment modalities were assessed with the Kruskal-Wallis test. Correlations between disease duration, time since treatment, and micronucleus frequency were evaluated using Spearman’s correlation coefficient, with a significance threshold set at P < 0.05.

## Results

**Table:** Table1. Baseline Characteristics of Newly Diagnosed and Treated Groups

**Characteristic**	**Newly Diagnosed (n=17)**	**Treated (n=17)**	**Sum**
**Sex, n (%)**			
**Male**	10 (58.8%)	7 (41.2%)	17 (50%)
**Female**	7 (41.2%)	10 (58.8%)	17 (50%)
**Age, mean (SD)**	60.24 (14.20)	60.71 (14.20)	-
**Lesion Location, n (%)**			
**Tongue**	10 (29.4%)	7 (20.5%)	17 (50%)
**Buccal Mucosa**	4 (11.7%)	4 (11.7%)	8 (23.5%)
**Mandibular Mucosa/Alveolar Ridge**	0	4 (11.7%)	4 (11.7%)
**Lower Lip**	1 (2.9%)	1 (2.9%)	2 (5.8%)
**Gingival Ulcers**	1 (2.9%)	1 (2.9%)	2 (5.8%)
**Maxilla**	1 (2.9%)	0	1 (2.9%)

**Figure-2:**
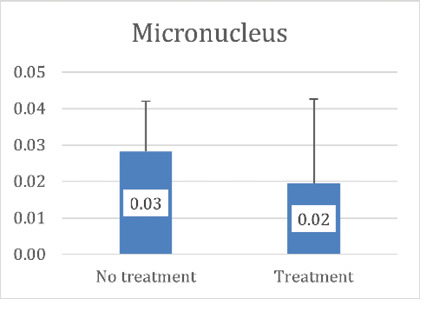
Figure 2. Distribution of the total number of micronuclei per 1000 cells

**Figure-3:**
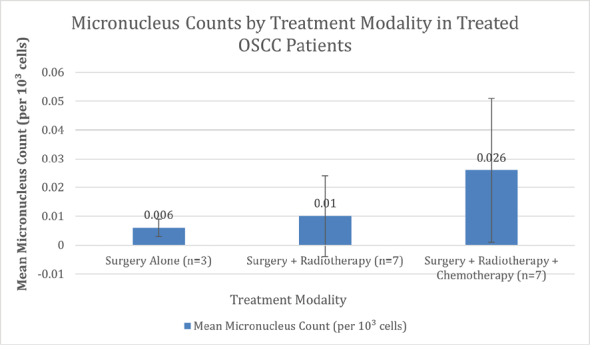
Figure 3. Micronucleus counts per 1000 cells in treated group based on the modality of treatment

This cross-sectional study compared 17 newly diagnosed and 17 treated patients with oral squamous cell carcinoma (OSCC) to evaluate demographic, clinical, and cytogenetic differences. The newly diagnosed group consisted of 10 males (58.8%) and 7 females (41.2%), while the treated group included 7 males (41.2%) and 10 females (58.8%). A chi-square test showed no significant difference in sex distribution between groups (p=0.303). The mean age was 60.24 years (SD=16.32) in the newly diagnosed group and 60.71 years (SD=14.20) in the treated group, with no significant difference (P=0.93). Lesion locations were distributed as follows: tongue (50%, n=17), buccal mucosa (23.5%, n=8), mandibular mucosa/alveolar ridge (11.7%, n=4), lower lip (5.8%, n=2), gingival ulcers (5.8%, n=2), and maxilla (2.9%, n=1). These results are summarized in Table-1.

T-test revealed no significant association between gender and micronucleus count in the newly diagnosed group (p=0.59). No such significant relationship was noticed in the treated group either (Mann-Whitney U=31.500, p=0.732). Across all patients, no significant correlation was observed between age and micronucleus count (Spearman’s ρ=0.228, p=0.194). The mean micronucleus count was significantly higher in the newly diagnosed group (0.028 ± 0.013 per 10³ cell) compared to the treated group (0.016 ± 0.020 per 10³ cell) (Mann-Whitney U=82.00, P=0.03), as shown in Figure-2.

Mean duration of disease in the newly diagnosed group was 5.94 months (SD=7.12), and average time since treatment in the treated group was 31.18 months (SD=43.2). In the newly diagnosed group, a slightly-significant trend suggested higher micronucleus counts with shorter disease duration (Spearman’s ρ=-0.471, P=0.056). In the treated group, no significant correlation was found between time since treatment and micronucleus count (Spearman’s ρ=0.188, P=0.469).

Treated Group-specific Comparisons

Among treated patients, micronucleus counts were compared across treatment modalities: surgery alone (n=3), surgery plus radiotherapy (n=7), and surgery plus radiotherapy and chemotherapy (n=7). Mean micronucleus counts were 0.006 per 10³ cell (SD=0.003) for surgery alone, 0.010 per 10³ cell (SD=0.014) for surgery plus radiotherapy, and 0.026 per 10³ cell (SD=0.025) for surgery plus radiotherapy and chemotherapy. The Kruskal-Wallis test showed no significant differences among treatment modalities (H=2.446, P=0.294). These findings are detailed in Figure-3.

## Discussion

The findings of this study indicated that the micronucleus count in buccal mucosal samples from treated patients with OSCC was significantly lower than that in newly diagnosed patients. The micronucleus count showed no significant correlation with the type of treatment received.

Genotoxins, radiation, chemicals, and congenital defects in DNA repair mechanisms can cause genetic damage [10]. Micronuclei are formed in cells exposed to genotoxic agents. Studies have shown that micronucleus count increases in cytological samples obtained from the buccal mucosa of smokers [7], hookah users [11], and patients with OSCC [2]. The micronucleus count has also been reported to be higher in patients with leukoplakia compared to healthy individuals; however, it is even higher in those with OSCC than in leukoplakia cases [9]. The study by Chaudhary et al. yielded similar results regarding the application of micronucleus count in OSCC patients, showing a significant difference between patients and healthy control groups [3]. These findings suggest that the micronucleus count in cytologic samples from buccal mucosa may be used as a biomarker to study cytogenetic damage in patients with pre-cancerous and cancerous lesions.

Previous studies have demonstrated that micronucleus count correlates with the histopathologic grade of OSCC [12]. Additionally, micronucleus count has been reported to be higher in clinical stage IV than stage III [2]. Carvalho et al. reported increased micronucleus frequency in T3 and T4 stages compared to T1 and T2 in oral and oropharyngeal carcinomas [13]. The present study also showed that micronucleus count in OSCC patients was significantly higher than in treated individuals. These findings align with our study, indicating that OSCC is associated with increased micronucleus formation, and that disease grade and stage influence micronucleus count, while patient recovery leads to a decrease in micronucleus numbers.

In this study, micronucleus count was not related to the type of treatment. The study by Tak et al. found that the average micronucleus count in buccal mucosal cells of oral cancer patients undergoing radiotherapy was significantly higher than in healthy controls, although it was not associated with radiation dose [14]. Similarly, Minicucci et al. reported that in head and neck cancer patients treated with radiotherapy, micronucleus counts increased during treatment but returned to baseline levels within 30 to 140 days after treatment completion [15].

Although the present study did not compare micronucleus count across specific treatment types, it did show a reduction in micronucleus count following treatment. Given previous studies by Tak and Minicucci, which demonstrated the clastogenic effects of radiotherapy, further research is necessary to clarify the influence of treatment type on micronucleus count.

Consistent with previous researches, the current study indicates that micronucleus count can be used as a screening test to monitor OSCC progression [16][17].

The findings also showed an inverse relationship between disease duration and micronucleus count: the shorter the duration of illness, the higher the micronucleus count. However, time elapsed since treatment was not significantly associated with micronucleus count. No previous study was found examining micronucleus count in relation to disease duration or post-treatment time. Although this effect requires further study, it seems that it could be due to the natural exfoliation and turnover of damaged epithelial cells, which gradually removes micronucleated cells from the mucosal surface. Additionally, spontaneous apoptosis may eliminate severely damaged cells [18], and clonal selection may favor more stable cell populations.

In this study, only patients who had completed treatment at least six months prior were included in the treatment group, and long-term follow-up of treated individuals was not feasible. This was the main limitation of the present study. It is recommended that future researches investigate the screening value of micronucleus count in patients experiencing recurrence of OSCC.

## Conclusion

Findings of present study suggests that micronucleus count can be used as a tool to monitor the success of treatment and predict the recovery process in patients with OSCC.

## Conflict of Interest

The authors declared no conflict of interest.

## References

[R1] Proia NK, Paszkiewicz GM, Nasca MA, Franke GE, Pauly JL (2006). Smoking and smokeless tobacco associated human buccal cell
mutations and their association with oral cancer a review. Cancer Epidemiol Biomarkers Prev.

[R2] Palve DH, Tupkari JV (2008). Clinicopathological correlation of micronuclei in oral squamous
cell carcinoma by exfoliative cytology. J Oral Maxillofac Pathol.

[R3] Chaudhary M, Venkatapathy R, Oza N, Prashad KV, Malik S (2017). Evaluation of Micronuclei in Oral Squamous Cell Carcinoma: A
Cytological Study. Int J Oral Care Res.

[R4] Tolbert PE, Shy CM, Allen JW (1992). Micronuclei and other nuclear anomalies in buccal smears: Methods
development. Mutat Res.

[R5] Stich HF, Rosin MP, Vallejera MO (1984). Reduction with vitamin A and betacarotene administration of
proportion of micronucleated buccal mucosal cells in Asian betal nut and
tobacco chewers. Lancet.

[R6] Jalayer Naderi, Pour Pasha (2017). Comparison of cytotoxic effect of cigarette and waterpipe smoking
on human buccal mucosa. Int J Prev Med.

[R7] Farhadi S, Jahanbani J, Sadri D, Moridani SG (2015). The micronucleus assessment of buccal mucosa: A noninvasive
method in screening of smokers potentially exposed to oral cancer. IJBPAS.

[R8] Nersesyan AK, Vardazaryan NS, Gevorgyan AL, Arutyunyan RM (2002). Micronucleus level in exfoliated buccal mucosa cells of cancer
patients. Arch Oncol.

[R9] Khanna S, Purwar A, Singh NN, Sreedhar G, Singh S, Bhalla S (2014). Cytogenetic biomonitoring of premalignant and malignant oral
lesions by micronuclei assessment: A screening evaluation. European Journal of General Dentistry.

[R10] Sivasankari P, Kaur S, Reddy KS, Vivekanandam S, Rao RK (2008). Micronucleus index: An early diagnosis in oral carcinoma. J Anat Soc India.

[R11] Jalili S, Naderi NJ (2022). Comparison of repair index in cigarette and waterpipe smokers: A
biomonitoring assessment using human exfoliated buccal mucosa cells. International Journal of Preventive Medicine.

[R12] Kumar V, Rao NN, Nair NS (2000). Micronuclei in oral squamous cell carcinoma: A marker of
genotoxic damage. Indian J Dent Res.

[R13] deCarvalho MB, Ramirez A, Gattás GJ, Guedes AL, Amar A, Rapoport A, et al (2002). Relationship between the outcome and the frequency of micronuclei
in cells of patients with oral and oropharyngeal carcinoma. Rev Assoc Med Bras.

[R14] Tak A, Metgud R, Astekar M, Tak M (2014). Micronuclei and other nuclear anomalies in normal human buccal
mucosa cells of oral cancer patients undergoing radiotherapy: a field effect. Biotech Histochem.

[R15] Minicucci EM, Kowalski LP, Maia MA, Pereira A, Ribeiro LR, de Camargo, Salvadori DM (2005). Cytogenetic damage in circulating lymphocytes and buccal mucosa
cells of headandneck cancer patients undergoing radiotherapy. J Radiat Res.

[R16] Sangle VA, Bijjaragi S, Shah N, Kangane S, Ghule HM, Rani SA (2016). Comparative study of frequency of micronuclei in normal,
potentially malignant diseases and oral squamous cell carcinoma. J Nat Sc Biol Med.

[R17] Kiran K, Agarwal P, Kumar S, Jain K (2018). Micronuclei as a predictor for oral carcinogenesis. J Cytol.

[R18] Driessens G, Harsan L, Robaye B, Waroquier D, Browaeys P, Giannakopoulos X, Velu T, Bruyns C (2003). Micronuclei to detect in vivo chemotherapy damage in a p53
mutated solid tumour. Br J Cancer.

